# The Decline of Physical Activity with Age in School-Aged Children with Cerebral Palsy: A Single-Center Cross-Sectional Observational Study

**DOI:** 10.3390/jcm12134548

**Published:** 2023-07-07

**Authors:** Jinuk Lee, Min-Hwa Suk, Soojin Yoo, Jeong-Yi Kwon

**Affiliations:** 1 Department of Physical & Rehabilitation Medicine, Samsung Medical Center, Sungkyunkwan University School of Medicine, Seoul 06351, Republic of Korea; jinuk8780.lee@samsung.com; 2 Department of Physical Education, Hanyang University, Seoul 04763, Republic of Korea; minhwasuk7@gmail.com; 3 Department of Health and Human Performance, University of Texas, Rio Grande Valley, Edinburg, TX 78539, USA; soo.yoo@utrgv.edu

**Keywords:** children, cerebral palsy, physical activity, accelerometry, cardiorespiratory fitness, age

## Abstract

Maintaining physical activity is important for children with cerebral palsy (CP). This study examined whether age predicted habitual physical activity (HPA) or cardiorespiratory fitness (CRF) in school-aged children with CP and clarified the relationship between HPA and CRF. We utilized cross-sectional data from 39 children with CP (18 girls and 21 boys; mean age 7.44 years; mean body weight 24.76 kg; mean body mass index 15.97 kg/m^2^; hemiplegic or diplegic CP). The participants wore an accelerometer (ActiGraph) for five days to measure HPA, physical activity energy expenditure (kcal/kg/d), sedentary physical activity (%SPA), light physical activity, moderate-to-vigorous physical activity (%MVPA), and activity counts (counts/min). Participants underwent cardiopulmonary exercise tests on a treadmill using a modified Naughton protocol. Linear regression and correlation analyses were performed. *p*-value (two-tailed) < 0.05 was considered statistically significant. Age was positively associated with SPA. MVPA negatively correlated with resting heart rate (HR), and activity counts were negatively correlated with resting HR. In conclusion, our study found strong evidence of a negative association between HPA and age in school-aged children with CP. It highlights the importance of creating and improving recreational opportunities that promote physical activity in all children with CP, regardless of whether they are considered therapeutic.

## 1. Introduction

Cerebral palsy (CP) is a group of movement and postural disorders caused by lesions in the developing human brain [1]. It is one of the most common neurodevelopmental disorders that cause motor disabilities in children, affecting approximately 2 per 1000 live births [2]. Children with CP have activity limitations due to motor disturbances accompanied by disturbances in sensation, cognition, communication, perception, behavior, epilepsy, and musculoskeletal problems [1].

In the healthy general population, those with higher levels of physical activity have improved cardiorespiratory fitness (CRF) [3], bone mineral density [4], and decreased risk of cardiovascular diseases [5]. CRF is a useful diagnostic and prognostic health indicator. Improved CRF is known to prevent or reduce cardiovascular morbidity and mortality in various patient populations [6]. The prevalence of cardiometabolic disease is higher in adults with CP than in those without [7]. The increased risk of chronic diseases in patients with CP is related to physical inactivity [8]. Therefore, efforts to elevate CRF in patients with CP are important for preventing chronic metabolic diseases relevant to CRF and maintaining a healthier status [9].

Physical activity is defined as “bodily movement produced regularly by the contraction of skeletal muscles that results in a substantial increase over resting energy expenditure” [10]. Lower motor capacity owing to physical disability is associated with lower Habitual Physical Activity (HPA) in children with CP [11,12]. Recently, accelerometers have been introduced to monitor, measure, and analyze the amount and intensity of HPA [13,14,15]. A multi-country study [16] that researched the time spent engaging in physical activity in healthy European school-aged children (*n* = 1025; mean age, 11.6 ± 0.9 years) found that 65% of the time spent at school was sedentary, and only 5% of the time was moderate-to-vigorous physical activity. However, children with CP are more sedentary throughout the day than is typical of developing healthy children of their age [17].

A systematic review [18] showed that there is a positive association between CRF and HPA in healthy adolescents. However, the authors mentioned that heterogeneity of the factors related to low CRF demonstrates the complexity of this topic and the need for further studies to obtain definitive conclusions. Recently, Silva et al. [19] reported a linear trend in the interaction between HPA and CRF in healthy children and adolescents throughout the 36-month study period. Ryan et al. [20] reported that reduced moderate-to-vigorous physical activity and increased sedentary behavior measured using accelerometers (RT3 model) were associated with elevated blood pressure (BP) in children with CP aged 6–17 years. They used shuttle-run tests to evaluate CRF in their study.

To date, few studies have investigated the relationship between age and HPA in children with CP. According to a cross-sectional study of healthy adolescents [21] (*n* = 24,025, aged 5.0–18.0 years; from 20 studies in 10 countries obtained from 2008 to 2010), both vigorous- and moderate-intensity physical activity recorded by accelerometers showed an age-related decline. Moreover, vigorous-intensity physical activity proportionally decreased compared with moderate-intensity physical activity. A similar tendency for age-related decline may exist in children with CP. One observational study [22] that focused on characteristics affecting the amount of ambulatory activity in children with CP used a StepWatch to measure HPA (*n* = 62, Gross Motor Function Classification System [GMFCS] levels I–III, aged 7–13 years). Older age, higher GMFCS level, and type of CP (diplegic or quadriplegic, except for hemiplegic CP) were associated with low ambulatory activity on school days, while older age, bilaterality, and no sports club participation were associated with lower ambulatory activity on weekends. According to a prospective cohort study by Waltersson et al. [23], being more physically active during adolescence in children with CP (GMFCS I–V, *n* = 71) resulted in increased physical activity in adulthood. These findings suggest that lifestyle choices regarding physical activity are established at an early age in children with CP.

To the best of our knowledge, there are few reports on the effect of age on CRF in school-aged children with CP. In a prospective cohort study in Northern Ireland [24], children with CP (*n* = 184, mean age 10.8 ± 3.6 years, GMFCS levels I–IV) demonstrated an increase in oxygen costs through an oxygen consumption protocol after an average follow-up of 2 years, which means a deterioration of energy efficiency that demanded more energy expenditure while performing the same activity as their non-CP peers.

This study had two main objectives. First, we attempted to determine whether HPA measured using an accelerometer could be predicted by age in school-aged children with CP. Our null hypothesis was that HPA is not age-dependent in this population. We also sought to determine whether there is a correlation between CRF and HPA using a symptom-limited cardiorespiratory exercise test (CPX), known as the gold standard test for measuring CRF. Our second null hypothesis was that HPA is not correlated with CRF in school-aged children with CP.

## 2. Materials and Methods

### 2.1. Study Design

This cross-sectional analysis was conducted as a sub-study of our randomized controlled trial registered at ClinicalTrials.gov under the identifier NCT03870893. This study was conducted at the Samsung Medical Center in Seoul, Korea, between August 2017 and December 2019. A total of 39 ambulatory school-aged children with CP participated in this cross-sectional analysis. The data used in the study were collected from the baseline data of our randomized controlled trial before the intervention was administered. Children eligible to participate were identified using the institution’s database. The primary investigator screened children whose parents agreed to participate via telephone. The Institutional Review Board of the Samsung Medical Center approved this study (registration number: 2017-06-045).

### 2.2. Participants

Initially, 47 children with CP participated in this study. Because one child refused to undergo screening and seven children were unable to undergo CPX due to physical disability or psychological difficulties (fear), only 39 children were included in the subsequent analysis. Table 1 presents the characteristics of the participants. There were 18 girls and 21 boys, with a mean age of 7.44 ± 1.60 years. Age was defined as the chronological age. Body height was measured with the patient in the supine position. Body Mass Index (BMI; kg/m^2^) was calculated as body weight (kg) divided by the square of body height (m). GMFCS level I or II was used to categorize the participants’ levels of physical ability. The inclusion criteria were as follows: (1) diagnosis of CP, (2) GMFCS level I or II, (3) age between 6 and 12 years, and (4) body weight of <35 kg. The exclusion criteria were as follows: (1) selective dorsal rhizotomy or orthopedic surgery in the last year; (2) injection of botulinum toxin injection in the last 3 months; (3) poor visual acuity; (4) uncontrolled seizures; (5) hearing impairment; (6) severe intellectual disability; (7) hip dislocation; (8) Cobb angle > 30° in scoliosis; and (9) unhealed fracture.

### 2.3. Habitual Physical Activity

The HPA of the participants was measured using a GT3X triaxial accelerometer (ActiGraph, Pensacola, FL, USA). The GT3X accelerometer is a safe, reliable, and validated approach for measuring physical activity in children with CP, indicating that it is appropriate for rehabilitation research [25]. The participants wore the accelerometer around their waist with a belt for seven days, even while sleeping or resting. The device collected data between 10 a.m. and 8 p.m. When entering the water, the participants were instructed to remove the accelerometer. Activity count (counts/min) is an essential parameter indicating physical activity based on the vector magnitude recorded by the accelerometer during physical activity. The higher the activity count, the higher the intensity level [26]. Physical activity energy expenditure (PAEE) and the percentage of time spent in each level of physical activity (% moderate-to-vigorous physical activity, %MVPA; % light physical activity, %LPA; % sedentary physical activity, %SPA) were calculated from activity counts (counts/min) using ActiLife software (version 6.14.3, ActiGraph, Pensacola, FL, USA). PAEE (kcal/kg/d) was calculated using the Freedson model presented in the software [27]. Each cutoff value of physical activity was classified using Evenson’s cutoff value model, which has demonstrated high accuracy in determining physical activity levels [28]. In this study, 0–100 counts per minute indicated SPA, 101–2295 counts per minute indicated LPA, and 2296 or more counts per minute indicated MVPA.

### 2.4. Cardiorespiratory Fitness

To measure CRF, the participants performed symptom-limited CPX using a modified Naughton protocol. We used a submaximal test because the Bruce protocol, a commonly used method for measuring CRF [29], was too difficult for our study participants. The modified Naughton protocol has 2 min stages, starting at a lower metabolic equivalent of task (MET) workload and increasing gradually by 1 MET per stage [30]. The gradient ratio was increased by 3.5% every 2 min until the maximal effort was achieved. Occasionally, it decreased by 2.5% to 3%, but for the most part, the velocity either increased or remained consistent with each stage of the test. Ten stages were performed in 20 min, with a maximal gradient ratio of 17.5% (Supplementary Table S1). Electrocardiograms (ECG) were recorded during the test to determine whether an abnormal heart rhythm was present. ECG were performed using a Cardiac Stress Testing System (Q-stress; Mortara Instrument Inc., Milwaukee, WI, USA). CRF was estimated using a True One 2400 (Parvo Medics, Salt Lake City, UT, USA). All the children were verbally encouraged to continue the test as much as possible. The reasons for ending the test included breathing difficulties, lower limb fatigue, exhaustion, increased heart rate, abnormal blood pressure responses, arrhythmias, increased chest pain, marked ST depression during the test, and refusal to continue. Exercise time (min), peak oxygen uptake (VO_2_ peak; mL/kg/min), MET (metabolic equivalent of task; kcal/kg/h), and respiratory exchange ratio (RER; ratio of produced VCO_2_ divided by consumed VO_2_) were determined and analyzed. After resting in a seated position for 30 min, the resting heart rate (HR) (beats/min), systolic blood pressure (SBP) (mmHg), and diastolic blood pressure (DBP; mmHg) were recorded. HR and BP were continuously monitored throughout CPX, and the maximum HR (beats/min), maximum SBP (mmHg), and maximum DBP (mmHg) during the exercise test (CPX) were recorded. To reduce the child’s psychological burden, one or more brief mock tests, a full explanation of the test procedure and meaning, and continued encouragement (before and during the test) were provided [31].

### 2.5. Motor Capacity

The participants underwent the Gross Motor Function Measure 66 (GMFM-66), timed up-and-go (TUG) test, and 6 min walk test (6 MWT) to measure motor capacity. Because it was difficult to repeat these tests multiple times, the statistical analysis was performed with the values measured only once. Furthermore, most children were familiar with these tests.

#### 2.5.1. Gross Motor Function Measure 66

The GMFM is a useful tool for assessing gross motor capacity in children with CP [32]. GMFM-66 scores were calculated from the GMFM-88 scores using the Gross Motor Ability Estimator. The GMFM-88 consists of 88 items across five dimensions: (A) lying and rolling (17 items); (B) sitting (20 items); (C) crawling and kneeling (14 items); (D) standing (13 items); and (E) walking, running, and jumping (24 items). Each item is scored on a 4-point scale.

#### 2.5.2. Timed Up-and-Go Test

The TUG test was used to measure the participants’ walking ability and functional movement capability, which have been shown to predict future health outcomes and quality of life of patients [33]. This test measures the time taken by the participant to rise from a chair, walk 3 m, turn 180°, walk 3 m again, and sit back in the chair [34]. The TUG test is a widely used, reliable, cost-effective, safe, valid, and time-efficient method for evaluating overall functional status [35]. The TUG test has been confirmed to be a reliable and responsive measure of mobility and balance in children with CP. The test–retest reliability of the TUG test was found to be high for GMFCS levels I (ICC = 0.970) and II (ICC = 0.981) in ambulatory children with CP aged between 3 and 10 years old [36].

#### 2.5.3. Six min Walk Test

This study used the 6 MWT to measure the walking distance. Participants walked without assistance for 6 min on a flat, straight, and indoor surface of at least 30 m in length [37]. The 6 MWT is a reflection of activities of daily living. It is easy to perform, does not require specialized equipment, and is cost-effective [37,38,39]. The 6 MWT has been confirmed to be reproducible and valid in children with CP. The test–retest reliability of the 6 MWT was found to be good for GMFCS levels I and II (ICC = 0.80) for children with CP (mean age, 14.2 ± 2.0 years) [40]. The test was conducted by two physical therapists according to a standardized guide. The children were provided detailed explanations before the test and were encouraged to continue.

### 2.6. Statistical Analysis

Statistical Package for the Social Sciences (SPSS) version 27.0 (IBM, Armonk, NY, USA) was used for the statistical analysis of the collected data; we set the Type 1 error rate at 5% and power at 80%. Using the Shapiro–Wilk test, we found that the HPA, CRF, and motor capacity data showed a normal distribution. After adjusting for sex, height, body weight (kg), BMI (kg/m^2^), and GMFCS score, a linear regression analysis was performed to examine whether age was associated with HPA, CRF, or motor capacity. A partial correlation analysis was also performed between the HPA and CRF to clarify their relationship. No data were missing. The results were considered statistically significant when the *p*-value (two-tailed) was <0.05.

### 2.7. Sample Size Calculation

Upon retrospective calculation, the total sample size required in our study was determined to be 29 in each group, with a Type 1 error rate of 0.05, Type 2 error rate of 0.2, and an expected correlation coefficient of 0.5. The sample size calculation for the original study (ClinicalTrial.gov, NCT03870893) has been previously described [41].

## 3. Results

### 3.1. HPA, CRF, and Motor Capacity

The means and SDs of HPA, CRF, and motor capacity variables were as follows: exercise time (min), 11.54 ± 3.40; VO_2_ peak (mL/kg/min), 25.50 ± 3.58; MET (kcal/kg/h), 7.29 ± 1.03; RER, 0.98 ± 0.05; resting HR (beats/min), 89.92 ± 12.64; resting SBP (mmHg), 101.67 ± 9.39; resting DBP (mmHg), 67.46 ± 8.47; maximal HR (beats/min), 167.41 ± 13.83; maximal SBP (mmHg), 122.56 ± 12.29; maximal DBP (mmHg), 67.26 ± 5.91; PAEE (kcal/kg/d), 4.49 ± 1.33; %SPA, 71.31 ± 5.42; %LPA, 23.40 ± 4.40; %MVPA, 5.29 ± 2.21; activity count (counts/min), 923.86 ± 232.18; TUG (s), 8.11 ± 2.02; 6 MWT (m), 376.62 ± 76.21; GMFM-66, 83.83 ± 10.74 (Table 2).

### 3.2. Linear Regression Analysis

According to the linear regression analysis (Table 3), age was negatively associated with PAEE (β = −0.496, *p =* 0.028) and activity counts (β = −0.516, *p =* 0.023) and positively associated with SPA (β = 0.463, *p =* 0.030). However, age did not predict any CRF or motor capacity variables in this analysis.

### 3.3. Partial Correlation Analysis

According to the partial correlation analysis (Table 4), PAEE was negatively correlated with resting HR (ρ = −0.372, *p =* 0.033) and TUG (ρ = −0.361, *p =* 0.039); MVPA was negatively correlated with resting HR (ρ = −0.388, *p =* 0.026), maximal HR (ρ = −0.360, *p =* 0.039), and TUG (ρ = −0.385, *p =* 0.027); activity count was negatively correlated with resting HR (ρ = −0.354, *p =* 0.043) and resting DBP (ρ = −0.363, *p =* 0.038).

## 4. Discussion

Age was negatively associated with the amount of physical activity in school-aged children with CP. However, age could not predict the CRF or motor capacity of participants. Furthermore, we observed a positive association between higher levels of MVPA and PAEE and CRF. Therefore, efforts to increase HPA levels in school-aged children with CP should be emphasized.

Stability and decline in gross motor function among children and youths with CP (aged 2–21 years) were reported in a prospective longitudinal cohort study [42]. Furthermore, they found no evidence of a functional decline in children with GMFCS levels I and II. Our findings are also consistent with those of that study. We also found that age could not predict CRF in school-aged children with CP or GMFCS levels I and II. This result is thought to be due to the correlation between the CRF and motor capacity variables. We previously reported that CPX exercise time was strongly correlated with GMFM (Spearman’s rho = 0.714), moderately correlated with TUG (Spearman’s rho = 0.537), and moderately correlated with the 6 MWT (Spearman’s rho = 0.702) [31]. Peak oxygen uptake during CPX showed a weak correlation with GMFM and a moderate correlation with 6 MWT in school-aged children with CP.

To date, there has been limited information available regarding the relationship between the HPA and CRF in school-aged children with CP. We found PAEE was negatively correlated with resting HR (ρ = −0.372, *p =* 0.033), MVPA was negatively correlated with resting HR (ρ = −0.388, *p =* 0.026), and maximal HR (ρ = −0.360, *p =* 0.039), while activity count was negatively correlated with resting HR (ρ = −0.354, *p =* 0.043) and resting DBP (ρ = −0.363, *p =* 0.038). This result is consistent with those of previous studies. Ryan et al. [20] reported that vigorous activity (β = 0.339, *p <* 0.01), sustained moderate-to-vigorous activity (β = 0.250, *p <* 0.05), and total activity (β = 0.238, *p <* 0.05) were associated with the level achieved in the shuttle run test after adjusting for age, sex, and GMFCS levels in ambulatory children with CP (aged 6–17 years, GMFCS levels I and II). The shuttle run test score was negatively associated with BMI (r^2^ = −0.451, *p <* 0.01), waist circumference (r^2^ = −0.560, *p <* 0.001), waist-to-height ratio (r^2^ = −0.560, *p <* 0.001), and systolic blood pressure (r^2^ = −0.306, *p <* 0.05) after adjusting for age, sex, and GMFCS score. They also reported that elevated BP values were associated with reduced time spent in moderate-to-vigorous, vigorous, and total activities as well as increased time spent in sedentary behavior [43]. Our study has several strengths, one of which is the use of a symptom-limited CPX. This method is considered the gold-standard approach for assessing CRF.

The decline in HPA levels at school age can be one of the contributing factors to the stagnation of motor capacity and CRF improvement in adolescence. It is possible there may be a non-visible deterioration that is not observed during school age. As mentioned previously, participants in a prospective cohort study in Northern Ireland [24] showed a decline in energy efficiency as their age increased. Interestingly, significant improvements in mean raw scores for GMFM, Pediatric Evaluation of Disability Inventory (PEDI), and Lifestyle Assessment Questionnaire-Cerebral Palsy (LAQ-CP) were recorded in this cohort. As CRF is not easily detectable in a typical setting, it can be overlooked, and the significance of CRF in early life can be underestimated.

In the general older adult population, an increase in physical activity levels is associated with a decrease in cardiovascular disease (CVD) risk factors, including blood pressure and insulin sensitivity [44]. In a prospective longitudinal cohort study (*n* = 630, aged 8–10 years), adiposity was the main predictor of insulin sensitivity after two years. Moreover, higher physical activity and less sedentary time appeared to benefit insulin sensitivity by lowering adiposity [45]. According to a recent systematic review, hypertension, obesity, and being overweight are significant risk factors for CVD in adults with CP [46]. Ryan et al. [47] reported a significant relationship between reduced physical activity and increased CVD risk in adults with CP. Therefore, it is reasonable to consider that the prevention of CVD would increase with the level of activity in school-going children with CP as well as adults. Additional longitudinal studies on HPA and CVD risk factors are needed in young populations with CP.

In a systematic review [48], a structured exercise program and an online behavioral program helped increase the HPA of children with CP; however, this positive effect was not maintained after the program was terminated. Therefore, it is necessary to develop an optimized exercise and physical activity behavior change program that can be used in the long term to increase HPA. Another systematic review [49] showed that cardiorespiratory exercise training may improve aerobic fitness, although the available evidence is limited. In one randomized clinical trial [50] of spastic patients with CP (*n* = 57; mean age, 20 ± 3 years; GMFCS levels I–IV), lifestyle intervention with counseling was performed in the intervention group for six months. Significant reductions in total cholesterol, VO2 peak, and SBP were observed in the intervention group compared with the control group. However, in another clinical trial [51] (*n* = 49, aged 7–13 years, GMFCS levels I–III), in which a home-based physiotherapy program with counseling was administered to the intervention group for six months, there was no significant difference in the outcomes of physical performance, aerobic capacity, or GMFM-66 between the intervention and control groups. Although controversial, active intervention is required to improve CRF in children with CP, and related research should continue.

As mentioned earlier, a study found that ambulatory activity decreases as the age of children with CP increases [21]. The authors explained that ambulatory activity, such as step count, may appear to decrease with age due to the concurrent increase in body height and leg length. However, our study found that age was negatively associated with HPA, even after adjusting for body height and weight, which could act as confounders. We thought that as children aged, sedentary activities such as studying, online gaming, and using smartphones would increase rather than moderate or vigorous physical activities such as playing sports. As mentioned earlier, in children, the majority of school time comprises sedentary activities [16]. Thus, it is necessary to develop a program to promote physical activity in children with CP, as well as in healthy children. A recent study [52] that researched the barriers and facilitators of physical activity in children with CP reported that physical activity could be promoted when academic work and physical activity were seen as equally important school priorities. Therefore, sufficient physical education in school environments should be encouraged.

This study has several limitations. First, though the sample size is absolutely small, it is larger than the calculated sample size of the original study this study retrospectively proceeded. Too high a sample size can amplify the detection of differences; a prospective study would be further needed. Second, because children with CP cannot perform the cardiorespiratory exercise test maximally, the interpretation of the results can be altered, and their reproducibility can be low. Third, the inclusion of children with GMFCS levels I–II was necessary due to physical limitations that affected their ability to perform the test. Fourth, the limited generalizability of our results to a larger population with CP should be acknowledged, as our study exclusively recruited Korean school-going children with CP. Finally, for a more objective and structured study design and a more generalized conclusion, a comparison of the HPA between children with CP and generally healthy populations in similar age groups is also needed, which should be undertaken in the future as further research. The strength of this study is that HPA and CRF were measured using an accelerometer, direct PA measurement, and symptom-limited CPX. These established measurement tools enhance the reliability and validity of our findings. We found an age-dependent decline in HPA and its effect on cardiorespiratory fitness in school-aged children. Our hypothesis suggests that Asian children are expected to exhibit lower levels of physical activity compared to Western children. However, considering the findings from studies [53] indicating a decline in physical activity with age, it is likely that similar patterns may be observed across different ethnic groups. Therefore, conducting further research that includes diverse ethnic populations is necessary to gain a comprehensive understanding of the relationship between physical activity and age in children.

## 5. Conclusions

The key finding of this study is that there is a negative association between age and the amount of HPA in school-aged children with CP, indicating that as children with CP age, they tend to engage in more sedentary activity. Through this study, we observed a positive association between age and SPA and a negative association between age and PAEE, while MVPA showed a negative correlation with HR and maximal HR in children with CP during symptom-limited CPX. Additionally, activity count demonstrated a negative correlation with resting HR and DBP.

To date, the primary focus of interventions for children with CP has been to enhance their functional abilities through therapy sessions in clinical settings. However, this study suggests that it is important to consider interventions to improve physical fitness and physical activity levels in schools and at home as well. It is important to provide additional and improved recreational opportunities that promote high levels of HPA in all children, regardless of whether these activities are considered therapeutic. These endeavors will contribute to the overall well-being of children with CP by reducing the risk of developing cardiometabolic syndromes and enabling them to lead healthier lives.

## Figures and Tables

**Table 1:** Baseline characteristics of the participants.

Characteristics	Value (*n* = 39)
Age (years)	7.44 ± 1.60
Sex (girls/boys)	18/21
Height (cm)	124.06 ± 10.27
Weight (kg)	24.76 ± 5.14
Body Mass Index (kg/m^2^)	15.97 ± 2.02
Distribution (unilateral; hemiplegia/bilateral; diplegia)	19/20
GMFCS distribution (level I/level II)	21/18

Values are expressed as mean ± standard deviation.

**Table 2:** The mean and SDs of HPA, CRF, and motor capacity variables.

Variables	Mean ± SD	Variables	Mean ± SD
exercise time (min)	11.54 ± 3.40	maximal DBP (mmHg)	67.26 ± 5.91
VO_2_ peak (mL/kg/min)	25.50 ± 3.58	PAEE (kcal/kg/d)	4.49 ± 1.33
MET (kcal/kg/h)	7.29 ± 1.03	%SPA	71.31 ± 5.42
RER	0.98 ± 0.05	%LPA	23.40 ± 4.40
resting HR (beats/min)	89.92 ± 12.64	%MVPA	5.29 ± 2.21
resting SBP (mmHg)	101.67 ± 9.39	activity count (counts/min)	923.86 ± 232.18
resting DBP (mmHg)	67.46 ± 8.47	TUG (s)	8.11 ± 2.02
maximal HR (beats/min)	167.41 ± 13.83	6 MWT (m)	376.62 ± 76.21
maximal SBP (mmHg)	122.56 ± 12.29	GMFM-66	83.83 ± 10.74

MET, metabolic equivalent of task; RER, respiratory exchange ratio; HR, heart rate; SBP, systolic blood pressure; DBP, diastolic blood pressure; PAEE, physical activity energy expenditure; SPA, sedentary physical activity; LPA, light physical activity; MVPA, moderate-to-vigorous physical activity; TUG, timed up-and-go (test); 6 MWT, 6 min walk test; GMFM-66, gross motor function measure–66.

**Table 3:** Regression analysis results for factors associated with age and HPA, CRF, and motor capacity for children with cerebral palsy.

		HPA				
		PAEE (kcal/kg/d)	% SPA	% LPA	% MVPA	Activity Count (Counts/min)
Age (years)	B	−0.412	1.566	−1.223	−0.343	−74.694
	95% CI	(−0.777, −0.048)	(0.159, 2.972)	(−2.448, 0.002)	(−0.953, 0.268)	(−138.314, −11.074)
	β	−0.496	0.463	−0.447	−0.248	−0.516
	*p*	0.028 *	0.030 *	0.050	0.262	0.023 *
	R^2^	0.441	0.498	0.416	0.431	0.439
		CRF				
		Exercise time (min)	VO_2_ peak (mL/kg/min)	MET (kcal/kg/h)	RER	HR_rest (beats/min)
Age (years)	B	0.129	0.615	0.171	0.007	−2.035
	95% CI	(−0.639, 0.896)	(−0.480, 1.710)	(−0.142, 0.483)	(−0.007, 0.021)	(−6.468, 2.398)
	β	0.061	0.275	0.267	0.236	−0.258
	*p*	0.735	0.261	0.274	0.317	0.357
	R^2^	0.619	0.302	0.309	0.350	0.081
		CRF				
		SBP_rest (mmHg)	DBP_rest (mmHg)	HR_max (beats/min)	SBP_max (mmHg)	DBP_max (mmHg)
Age (years)	B	0.076	0.048	1.328	2.583	0.617
	95% CI	(−3.004, 3.155)	(−2.484, 2.580)	(−3.141, 5.797)	(−0.151, 5.317)	(−0.923, 2.157)
	β	0.013	0.009	0.154	0.339	0.167
	*p*	0.960	0.970	0.549	0.063	0.420
	R^2^	0.197	0.333	0.221	0.627	0.493
		Motor capacity TUG (s)	6 MWT (m)	GMFM-66		
Age (years)	B	0.004	−6.519	−0.089		
	95% CI	(−0.606, 0.615)	(−25.034, 11.997)	(−4.237, 0.058)		
	β	0.003	−0.137	1.054		
	*p*	0.989	0.478	0.056		
	R^2^	0.318	0.559	0.701		

B: unstandardized coefficient; β: standardized coefficient. CI, confidence interval; MET, metabolic equivalent of task; RER, respiratory exchange ratio; HR, heart rate; SBP, systolic blood pressure; DBP, diastolic blood pressure; PAEE, physical activity energy expenditure; SPA, sedentary physical activity; LPA, light physical activity; MVPA, moderate-to-vigorous physical activity; TUG, timed up-and-go (test); 6 MWT, 6 min walk test; GMFM-66, Gross Motor Function Measure 66. *: *p* <0.05

**Table 4:** Partial correlation analysis.

		Exercise Time (min)	VO_2_ Peak (mL/kg/min)	MET (kcal/kg/h)	RER	HR_Rest (beats/min)	SBP_Rest (mmHg)	DBP_Rest (mmHg)	HR_Max (beats/min)	SBP_Max (mmHg)	DBP_Max (mmHg)	TUG (s)	6MWT (m)	GMFM-66
PAEE (kcal/kg/d)	*ρ*	0.202	−0.045	−0.040	−0.059	−0.372	−0.114	−0.212	−0.323	−0.328	−0.037	−0.361	0.002	0.299
	*p*	0.261	0.802	0.823	0.744	0.033 *	0.526	0.237	0.067	0.062	0.837	0.039 *	0.993	0.091
%SPA	*ρ*	−0.158	−0.115	−0.115	0.218	0.294	0.140	0.268	0.218	0.064	−0.003	0.228	0.152	−0.180
	*p*	0.379	0.524	0.523	0.222	0.097	0.437	0.132	0.223	0.724	0.986	0.202	0.397	0.316
%LPA	*ρ*	0.083	0.162	0.159	−0.226	−0.144	−0.104	−0.230	−0.071	0.094	0.002	−0.070	−0.213	0.052
	*p*	0.644	0.368	0.378	0.206	0.423	0.566	0.198	0.696	0.603	0.993	0.699	0.233	0.774
%MVPA	*ρ*	0.197	−0.060	−0.053	−0.050	−0.388	−0.114	−0.156	−0.360	−0.335	0.004	−0.385	0.077	0.311
	*p*	0.271	0.740	0.770	0.782	0.026 *	0.526	0.386	0.039 *	0.056	0.981	0.027 *	0.670	0.078
Activity count (counts/min)	*ρ*	0.153	0.108	0.110	−0.210	−0.354	−0.203	−0.363	−0.312	−0.172	−0.097	−0.264	−0.243	0.252
	*p*	0.396	0.548	0.543	0.242	0.043 *	0.258	0.038 *	0.077	0.339	0.591	0.138	0.173	0.157

MET, metabolic equivalent of task; RER, respiratory exchange ratio; HR, heart rate; SBP, systolic blood pressure; DBP, diastolic blood pressure; PAEE, physical activity energy expenditure; SPA, sedentary physical activity; LPA, light physical activity; MVPA, moderate-to-vigorous physical activity; TUG, timed up-and-go (test); 6 MWT, 6 min walk test; GMFM-66, gross motor function measure—66. *: *p* <0.05

## Data Availability

The data supporting the findings of this study are available from the corresponding author upon request.

## Supplementary Materials

The following supporting information can be downloaded at: https://www.mdpi.com/article/10.3390/jcm12134548/s1. Table S1: Modified Naughton Protocol.
