# Supplementary material for: The Decline of Physical Activity with Age in School-Aged Children with Cerebral Palsy: A Single-Center Cross-Sectional Observational Study
Source: J Clin Med. 2023 Jul 7;12(13):4548. doi: 10.3390/jcm12134548 (PMC10342892; doi:10.3390/jcm12134548)
Supplement: Supplementary file 1 [file jcm-12-04548-s001.zip › jcm-2408424-supplementary.pdf]

Table S1. Modified Naughton Protocol.

Stage	min	km/h	%GR		Stage	min	km/h	%GR
**1**	2	1.6	0		**6**	12	4.8	7.5
**2**	4	2.4	0		**7**	14	4.8	10
**3**	6	3.2	3.5		**8**	16	4.8	12.5
**4**	8	3.2	7.0		**9**	18	4.8	15
**5**	10	3.2	10.5		**10**	20	4.8	17.5

GR: Gradient Ratio
